# Supplementary material for: Carbon Dioxide Adsorption over Activated Carbons Produced from Molasses Using H2SO4, H3PO4, HCl, NaOH, and KOH as Activating Agents
Source: Molecules. 2022 Nov 2;27(21):7467. doi: 10.3390/molecules27217467 (PMC9653830; doi:10.3390/molecules27217467)
Supplement: Supplementary file 1 [file molecules-27-07467-s001.zip › molecules-1984231-supplementary.pdf]

Supplementary

Carbon Dioxide Adsorption over Activated Carbons Produced from Molasses Using H_2SO_4, H_3PO_4, HCl, NaOH, and KOH as Activating Agents

Karolina Kiełbasa [1], Şahin Bayar [2], Esin Apaydin Varol [2], Joanna Sreńscek-Nazzal [1], Monika Bosacka [3], Piotr Miądlicki [1], Jarosław Serafin [4,*], Rafał J. Wróbel [1,*] and Beata Michalkiewicz [1]

[1] Faculty of Chemical Technology and Engineering, Department of Catalytic and Sorbent Materials Engineering, West Pomeranian University of Technology in Szczecin, Piastów Ave. 42, 71-065 Szczecin, Poland
[2] Faculty of Engineering, Deptarment of Chemical Engineering, Eskisehir Technical University, Eskisehir 26555, Turkey
[3] Faculty of Chemical Technology and Engineering, Department of Inorganic and Analytical Chemistry, West Pomeranian University of Technology in Szczecin, Piastów Ave. 42, 71-065 Szczecin, Poland
[4] Department of Inorganic and Organic Chemistry, University of Barcelona, Martí i Franquès, 1-11, 08028 Barcelona, Spain
* Correspondence: jaroslaw.serafin@qi.ub.es (J.S.); rafal.wrobel@zut.edu.pl (R.J.W.)

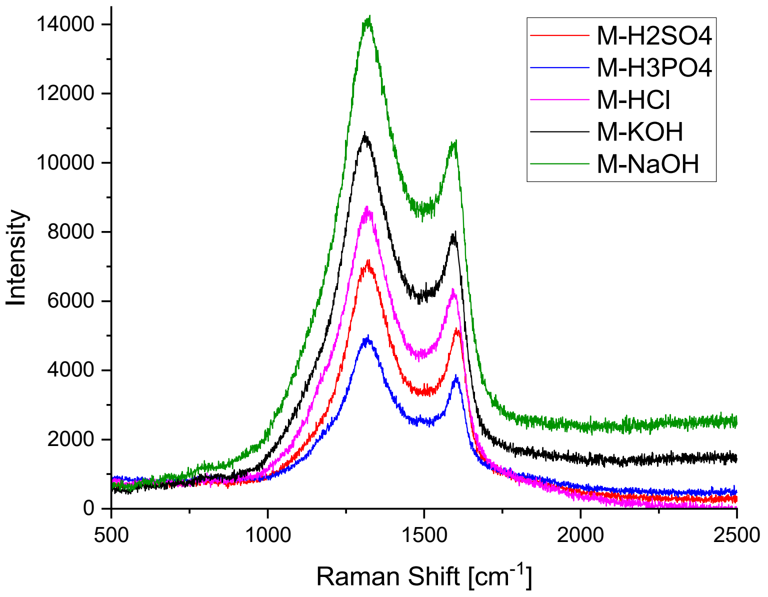

Figure S1. Raman spectra of the activated carbons produced from molasses

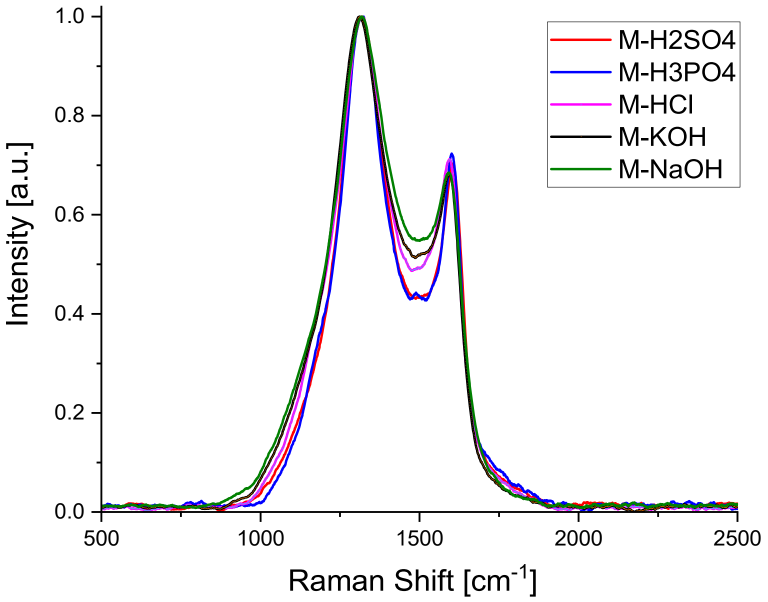

Figure S2. Raman spectra of the activated carbons produced from molasses after the smoothing, baseline subtracting and normalizing to the D band intensity

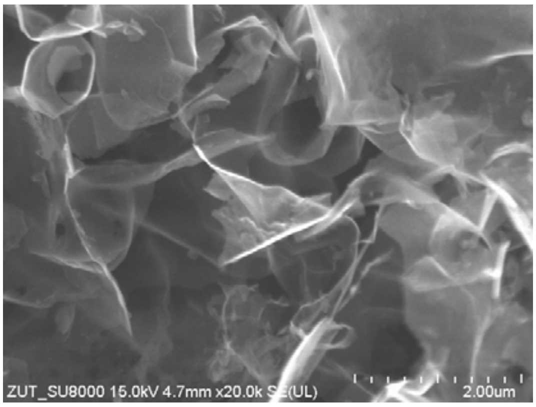

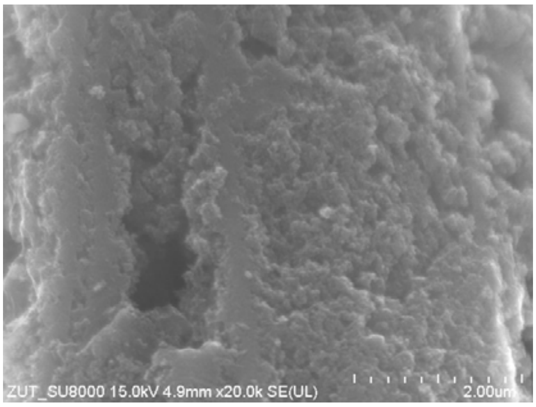

M-NaOH

M-H3PO4

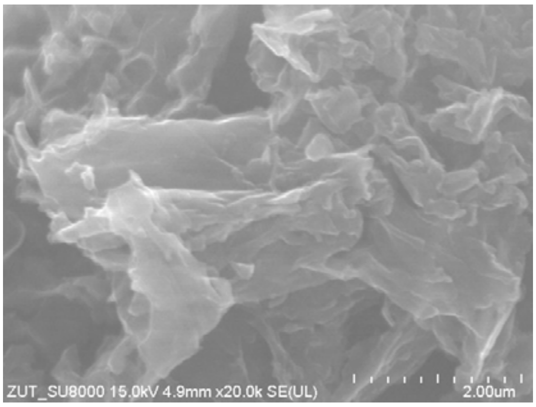

M-HCl

Figure S3. SEM pictures of the activated carbons produced from molasses

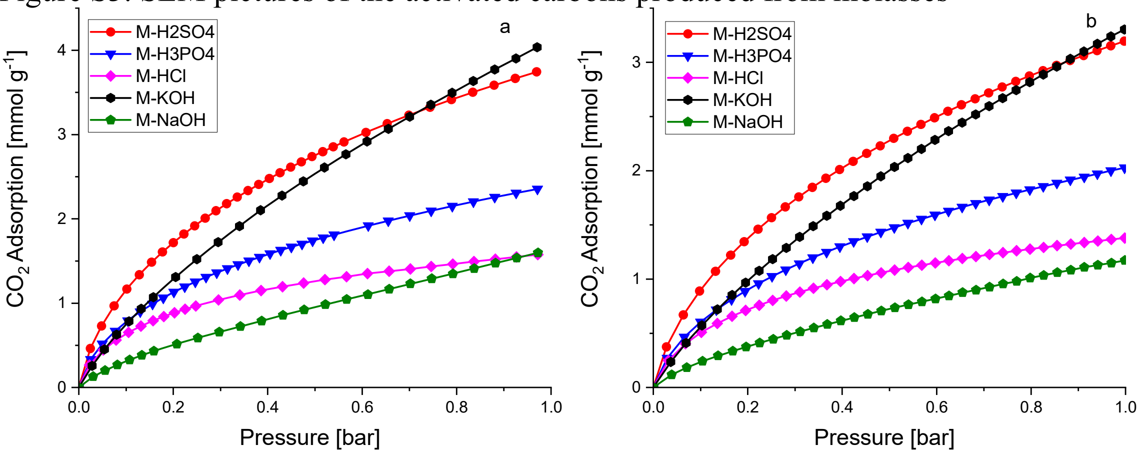

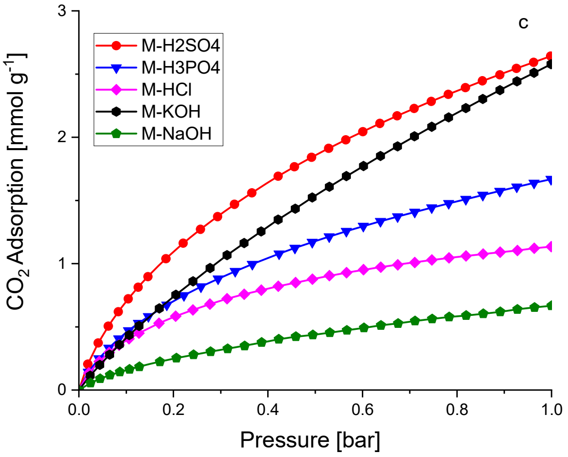

Figure S4. CO_2 adsorption over activated carbons produced from molasses at the temperatures of 10 ºC (a), 20 ºC (b), 30 ºC (c)

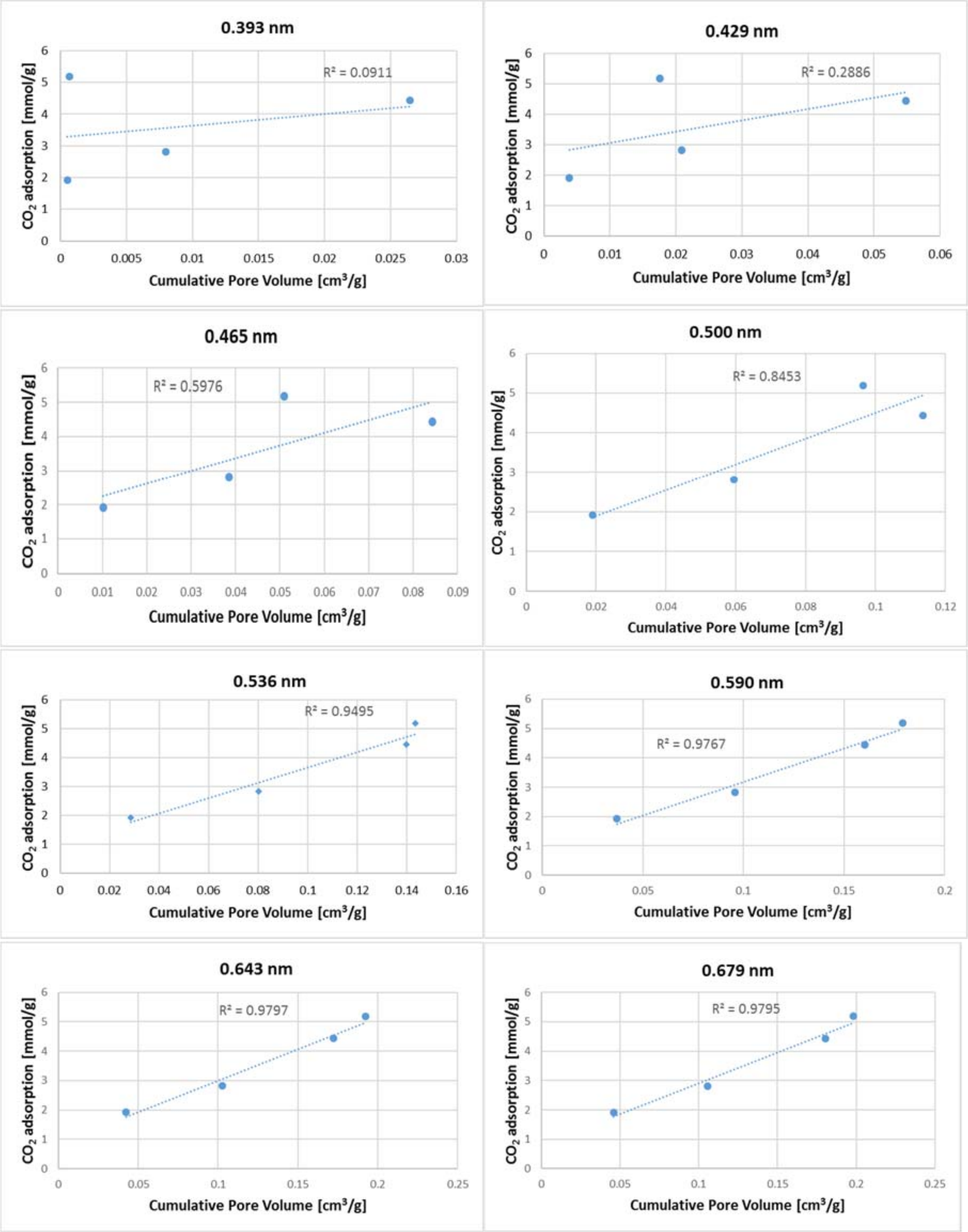
0.393 nm
R² = 0.0911
CO2 adsorption [mmol/g]
Cumulative Pore Volume [cm3/g]
0.429 nm
R² = 0.2886
CO2 adsorption [mmol/g]
Cumulative Pore Volume [cm3/g]
0.465 nm
R² = 0.5976
CO2 adsorption [mmol/g]
Cumulative Pore Volume [cm3/g]
0.500 nm
R² = 0.8453
CO2 adsorption [mmol/g]
Cumulative Pore Volume [cm3/g]
0.536 nm
R² = 0.9495
CO2 adsorption [mmol/g]
Cumulative Pore Volume [cm3/g]
0.590 nm
R² = 0.9767
CO2 adsorption [mmol/g]
Cumulative Pore Volume [cm3/g]
0.643 nm
R² = 0.9797
CO2 adsorption [mmol/g]
Cumulative Pore Volume [cm3/g]
0.679 nm
R² = 0.9795
CO2 adsorption [mmol/g]
Cumulative Pore Volume [cm3/g]

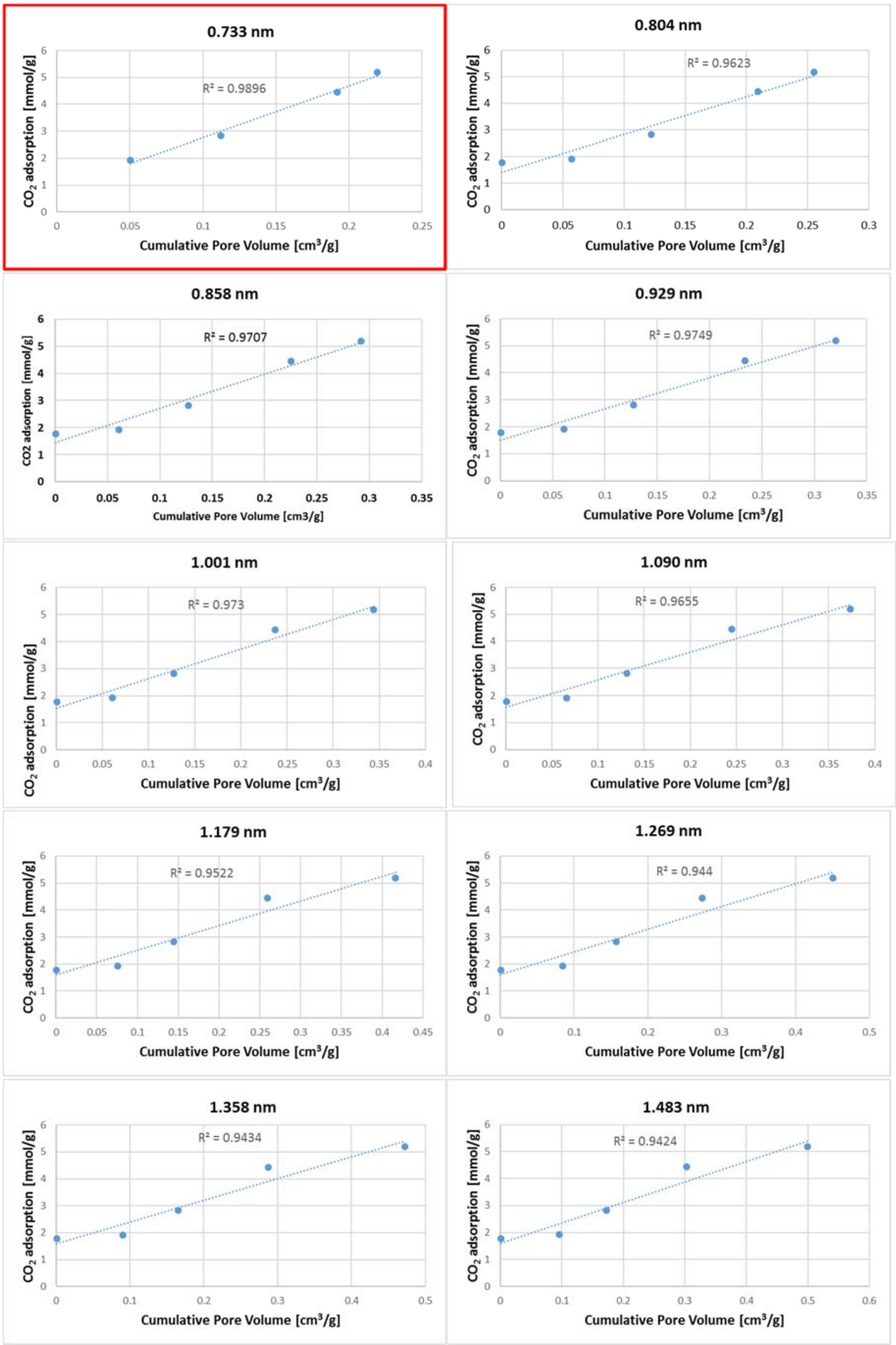
0.733 nm
R² = 0.9896
0.804 nm
R² = 0.9623
0.858 nm
R² = 0.9707
0.929 nm
R² = 0.9749
1.001 nm
R² = 0.973
1.090 nm
R² = 0.9655
1.179 nm
R² = 0.9522
1.269 nm
R² = 0.944
1.358 nm
R² = 0.9434
1.483 nm
R² = 0.9424
CO2 adsorption [mmol/g]
Cumulative Pore Volume [cm3/g]

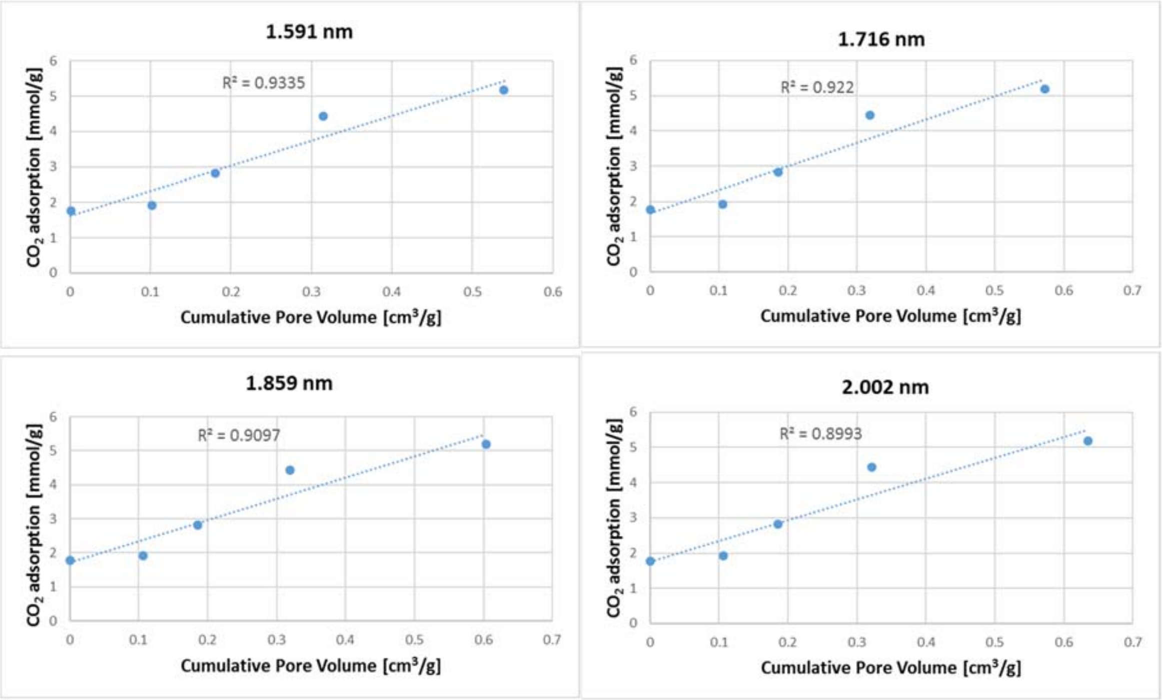

Figure S5. CO_2 adsorption at 1 bar and 0ºC as a function of the volume of pores which are equal to and below given diameter. The best fit was outlined in red.

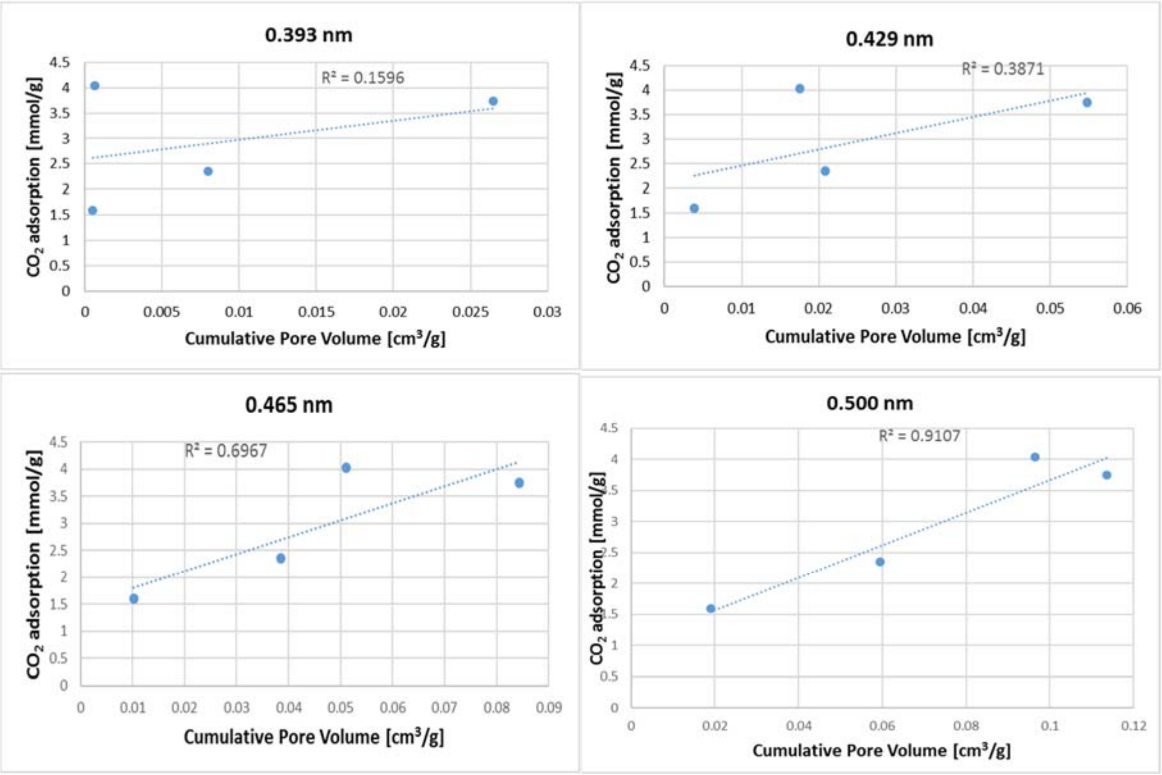

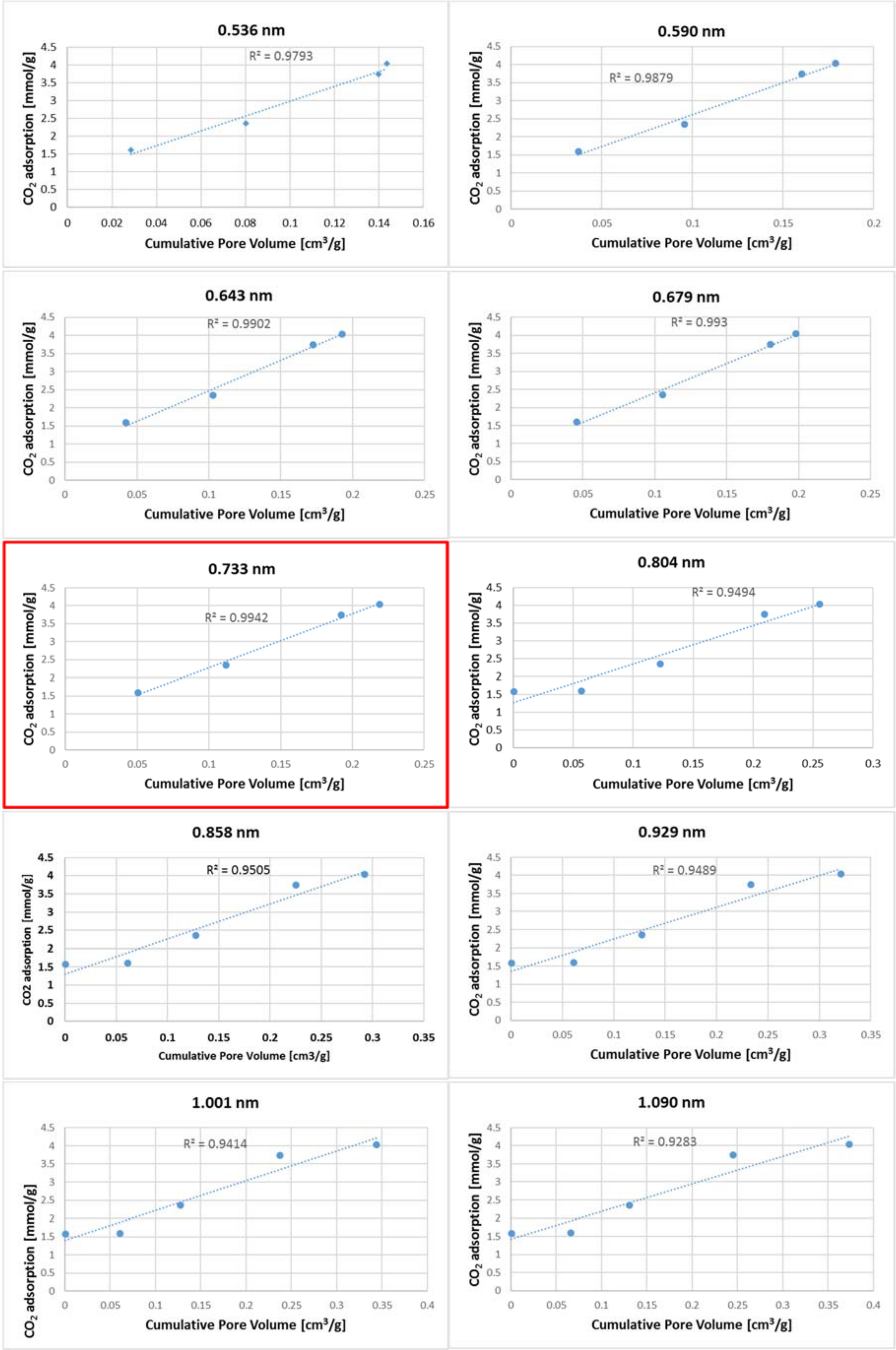

0.536 nm
R² = 0.9793
0.590 nm
R² = 0.9879
0.643 nm
R² = 0.9902
0.679 nm
R² = 0.993
0.733 nm
R² = 0.9942
0.804 nm
R² = 0.9494
0.858 nm
R² = 0.9505
0.929 nm
R² = 0.9489
1.001 nm
R² = 0.9414
1.090 nm
R² = 0.9283
CO2 adsorption [mmol/g]
Cumulative Pore Volume [cm3/g]

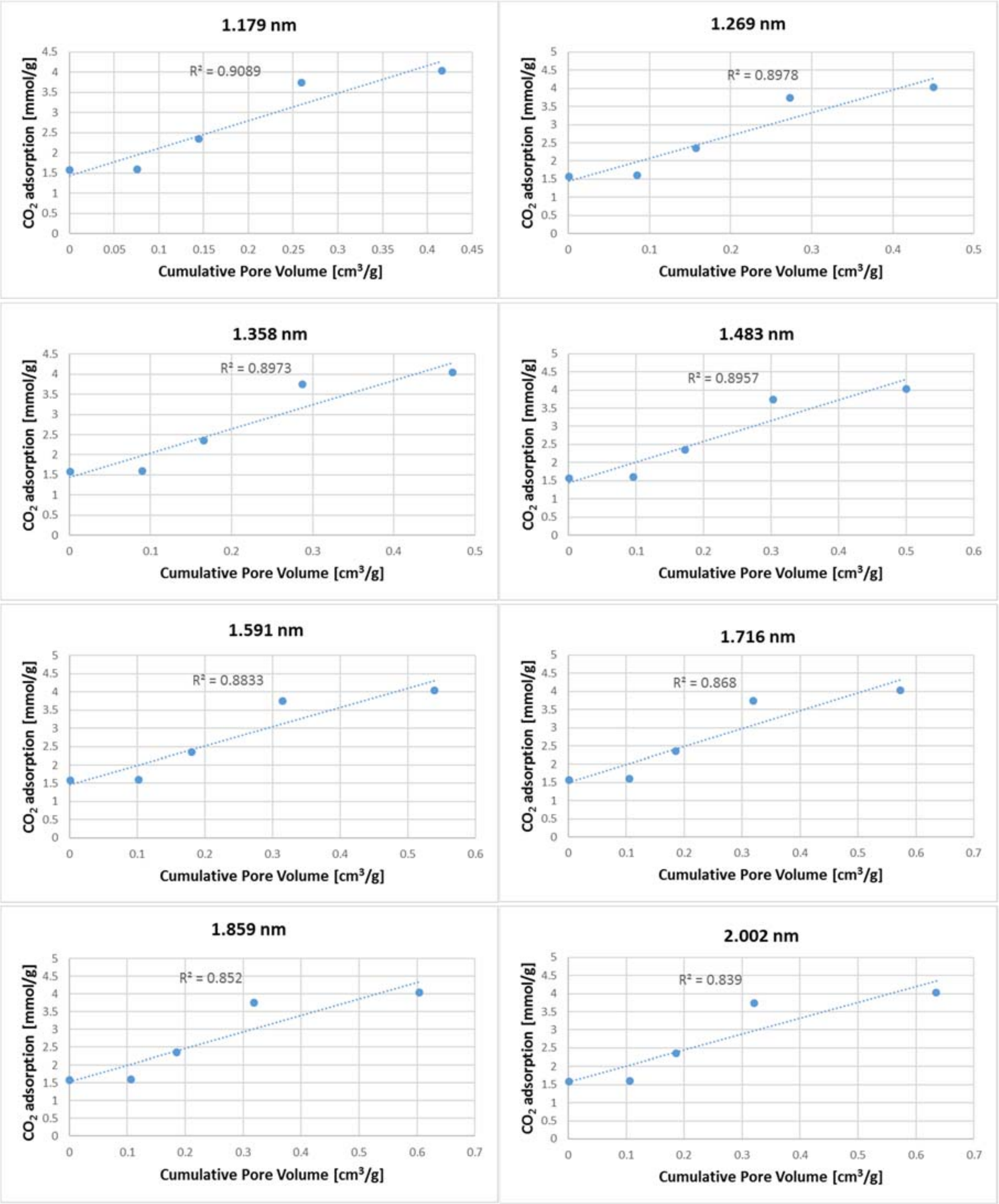

Figure S6. CO_2 adsorption at 1 bar and 10ºC as a function of the volume of pores which are equal to and below given diameter. The best fit was outlined in red.

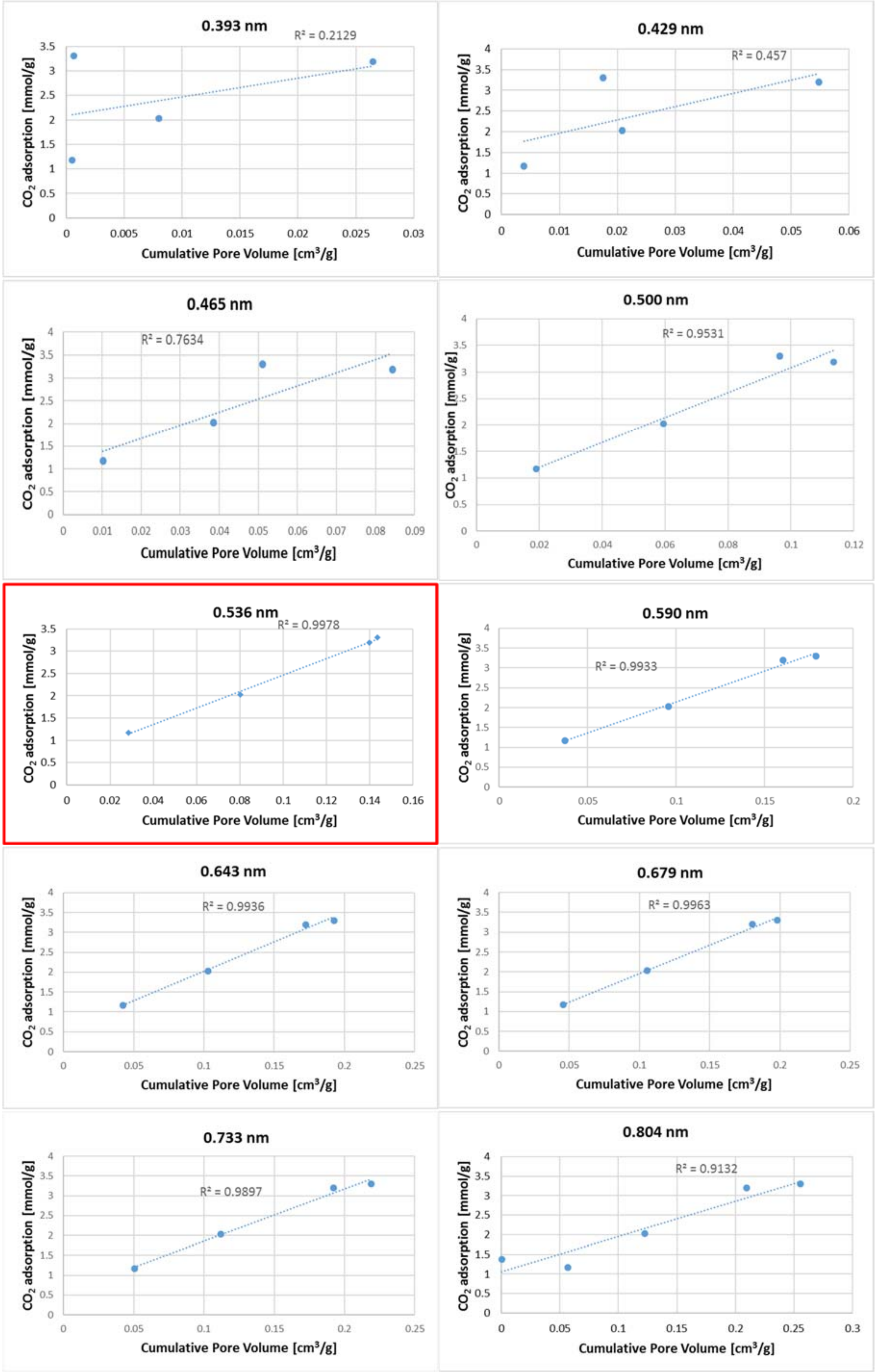
0.393 nm
R² = 0.2129
0.429 nm
R² = 0.457
0.465 nm
R² = 0.7634
0.500 nm
R² = 0.9531
0.536 nm
R² = 0.9978
0.590 nm
R² = 0.9933
0.643 nm
R² = 0.9936
0.679 nm
R² = 0.9963
0.733 nm
R² = 0.9897
0.804 nm
R² = 0.9132
CO2 adsorption [mmol/g]
Cumulative Pore Volume [cm3/g]

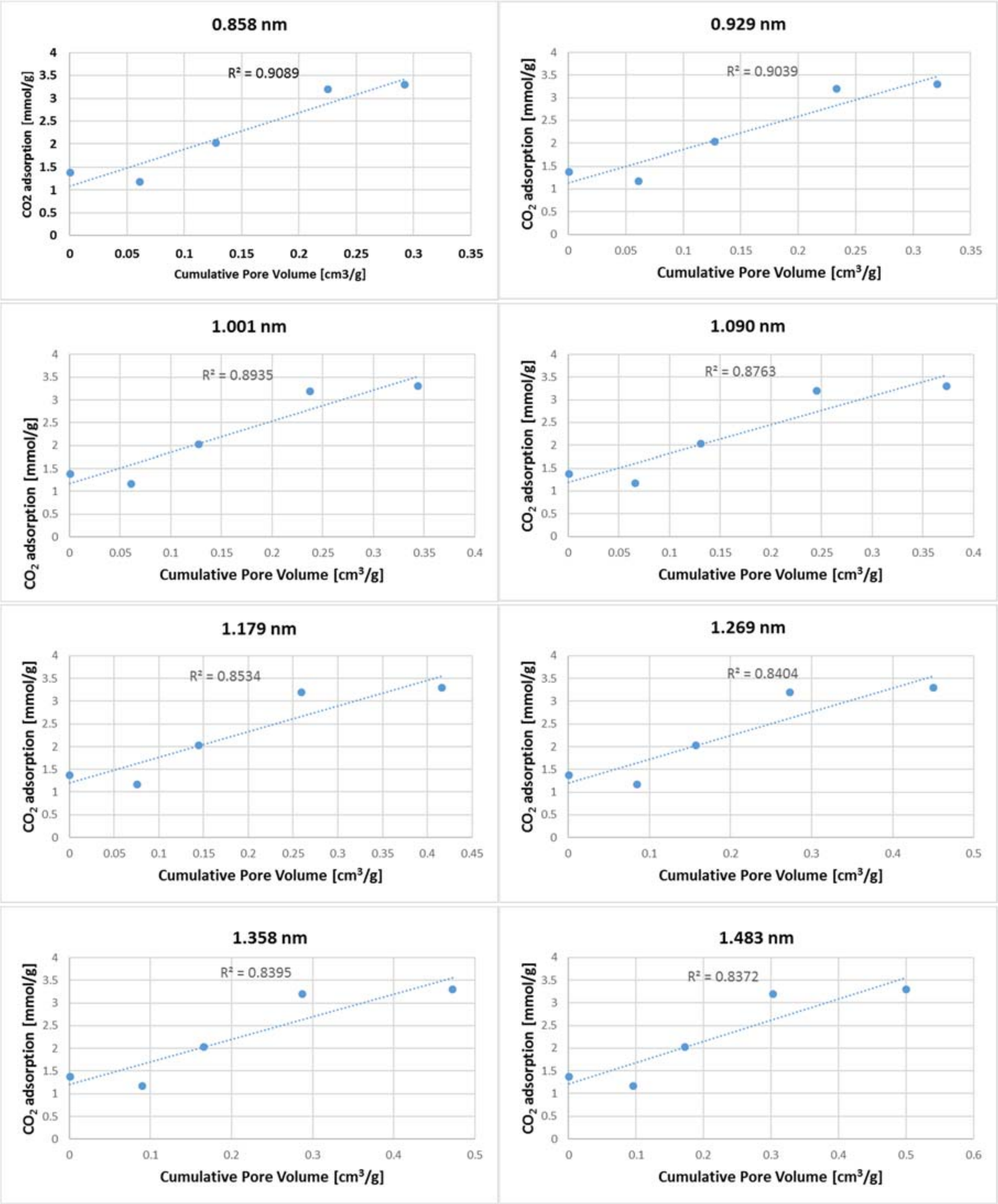
0.858 nm
R² = 0.9089
CO2 adsorption [mmol/g]
Cumulative Pore Volume [cm3/g]
0.929 nm
R² = 0.9039
CO₂ adsorption [mmol/g]
Cumulative Pore Volume [cm³/g]
1.001 nm
R² = 0.8935
CO₂ adsorption [mmol/g]
Cumulative Pore Volume [cm³/g]
1.090 nm
R² = 0.8763
CO₂ adsorption [mmol/g]
Cumulative Pore Volume [cm³/g]
1.179 nm
R² = 0.8534
CO₂ adsorption [mmol/g]
Cumulative Pore Volume [cm³/g]
1.269 nm
R² = 0.8404
CO₂ adsorption [mmol/g]
Cumulative Pore Volume [cm³/g]
1.358 nm
R² = 0.8395
CO₂ adsorption [mmol/g]
Cumulative Pore Volume [cm³/g]
1.483 nm
R² = 0.8372
CO₂ adsorption [mmol/g]
Cumulative Pore Volume [cm³/g]

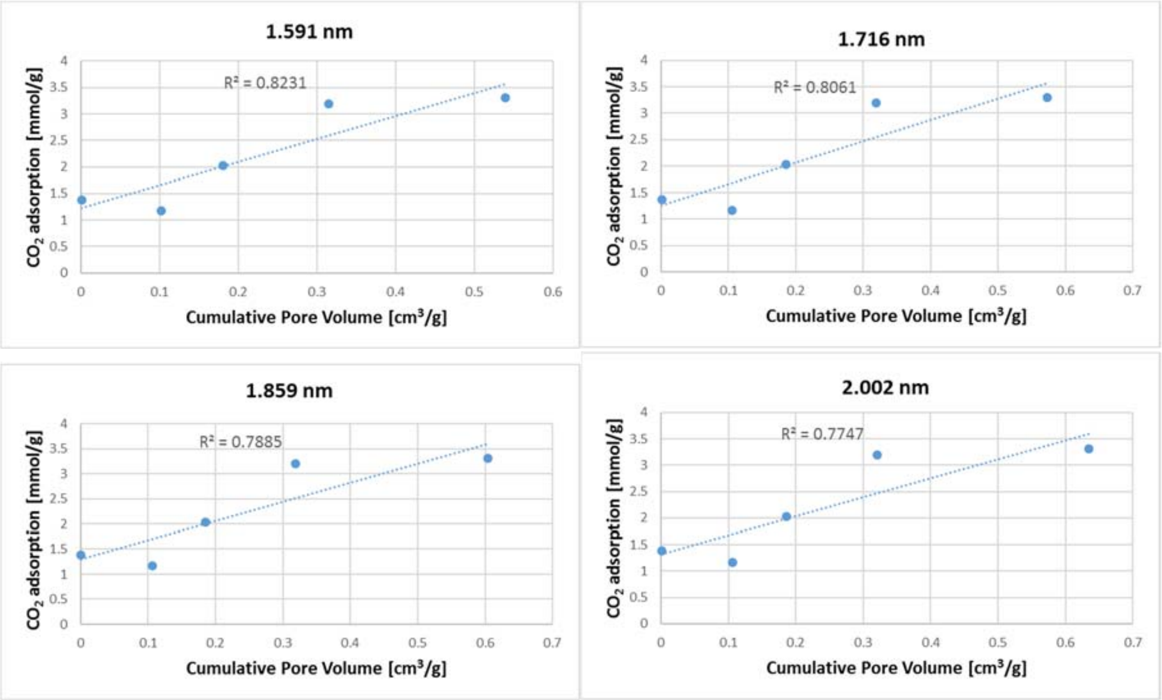

Figure S7. CO_2 adsorption at 1 bar and 20°C as a function of the volume of pores which are equal to and below given diameter. The best fit was outlined in red.

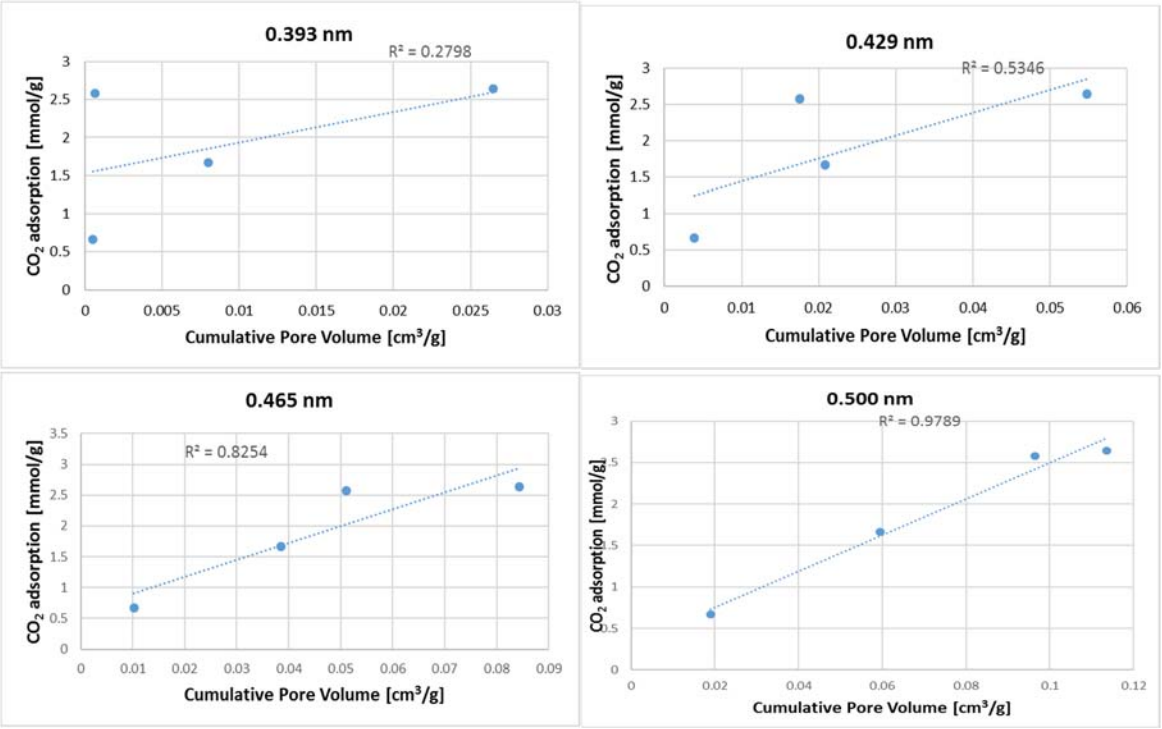

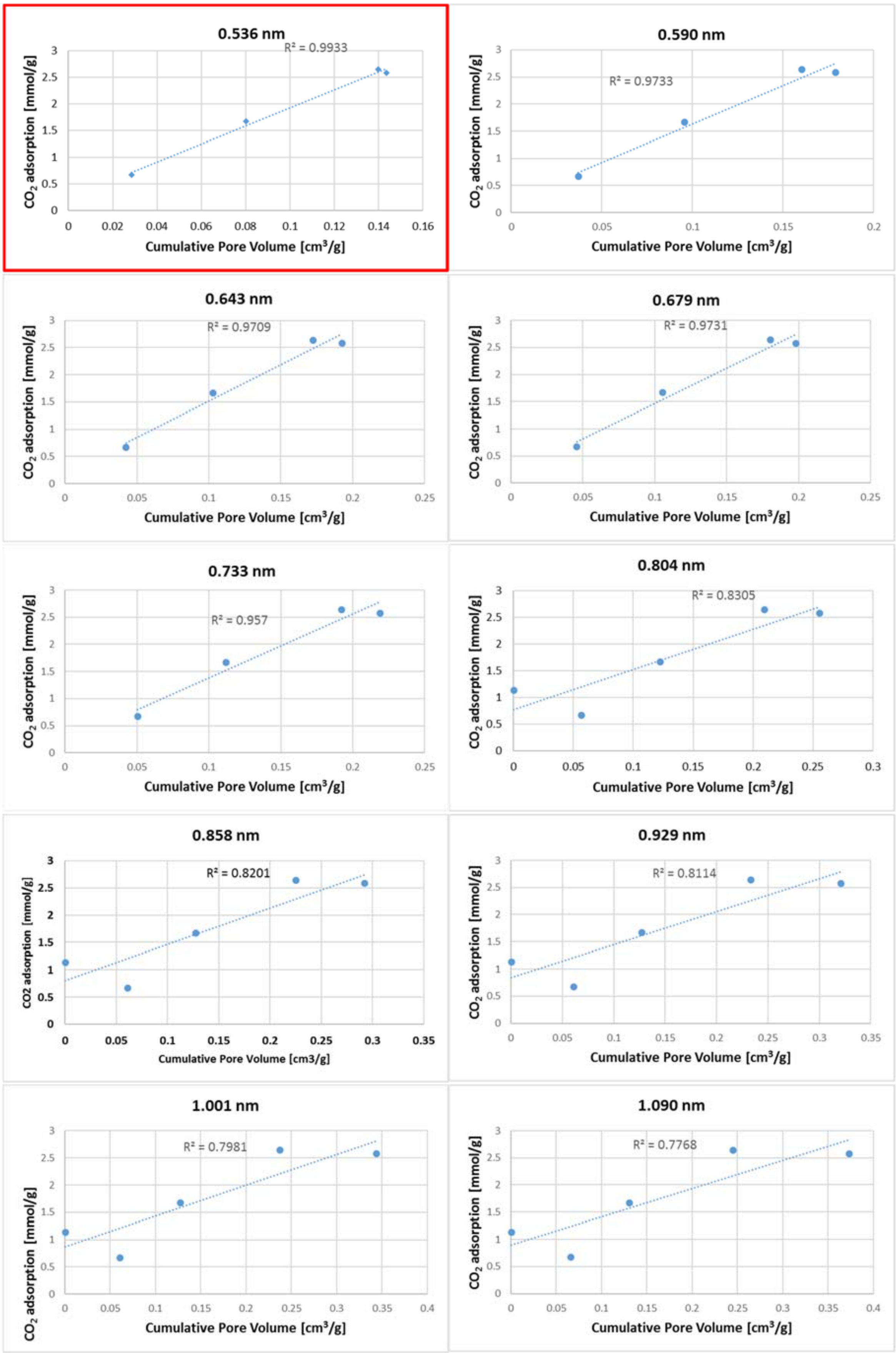

0.536 nm
R² = 0.9933
CO2 adsorption [mmol/g]
Cumulative Pore Volume [cm³/g]
0.590 nm
R² = 0.9733
CO2 adsorption [mmol/g]
Cumulative Pore Volume [cm³/g]
0.643 nm
R² = 0.9709
CO2 adsorption [mmol/g]
Cumulative Pore Volume [cm³/g]
0.679 nm
R² = 0.9731
CO2 adsorption [mmol/g]
Cumulative Pore Volume [cm³/g]
0.733 nm
R² = 0.957
CO2 adsorption [mmol/g]
Cumulative Pore Volume [cm³/g]
0.804 nm
R² = 0.8305
CO2 adsorption [mmol/g]
Cumulative Pore Volume [cm³/g]
0.858 nm
R² = 0.8201
CO2 adsorption [mmol/g]
Cumulative Pore Volume [cm3/g]
0.929 nm
R² = 0.8114
CO2 adsorption [mmol/g]
Cumulative Pore Volume [cm³/g]
1.001 nm
R² = 0.7981
CO2 adsorption [mmol/g]
Cumulative Pore Volume [cm³/g]
1.090 nm
R² = 0.7768
CO2 adsorption [mmol/g]
Cumulative Pore Volume [cm³/g]

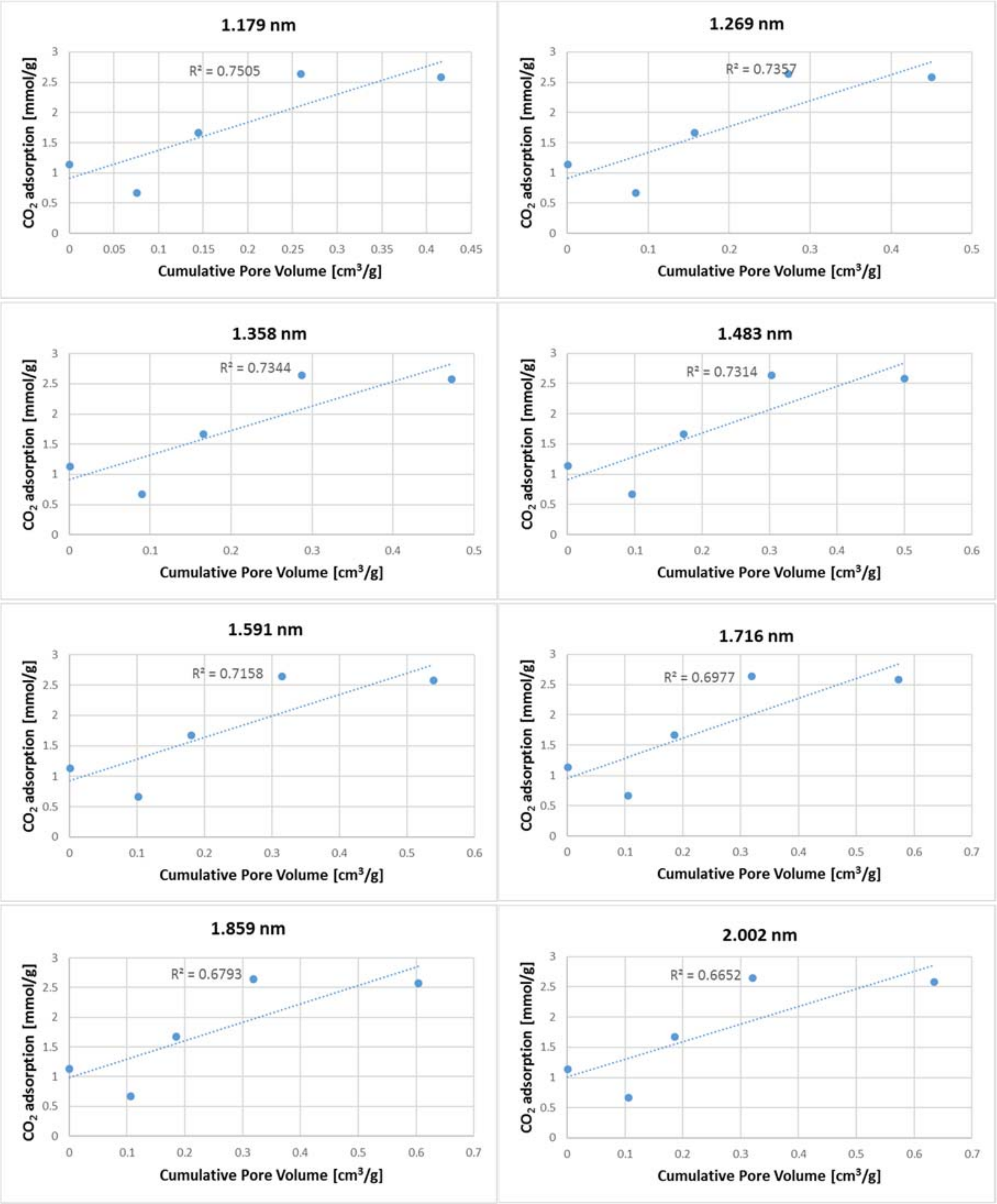

Figure S8. CO_2 adsorption at 1 bar and 30ºC as a function of the volume of pores which are equal to and below given diameter. The best fit was outlined in red.

Table S1. CO_2 adsorption at different temperatures at the pressure of 1 bar

Sample	CO_2 adsorption [mmol/g]			
	0 [ºC]	10 [ºC]	20 [ºC]	30 [ºC]
M-H2SO4	4.44	3.74	3.2	2.64

M-H3PO4	2.82	2.35	2.03	1.67
M-HCl	1.77	1.58	1.38	1.13
M-KOH	5.18	4.04	3.3	2.58
M-NaOH	1.92	1.6	1.17	0.67

Table S2. Parameters of the Toth model for CO_2 adsorption at different temperatures

	M-KOH			M-H2SO4		
Temperature [ºC]	q_{mT} [mmol/g]	b [bar^{-1}]	n	q_{mT} [mmol/g]	b [bar^{-1}]	n
0	30.10	0.452	0.522	23.80	3.708	0.305
10	28.12	0.346	0.526	21.13	2.513	0.324
20	27.12	0.257	0.532	19.13	1.679	0.341
30	24.57	0.202	0.540	17.75	1.265	0.343

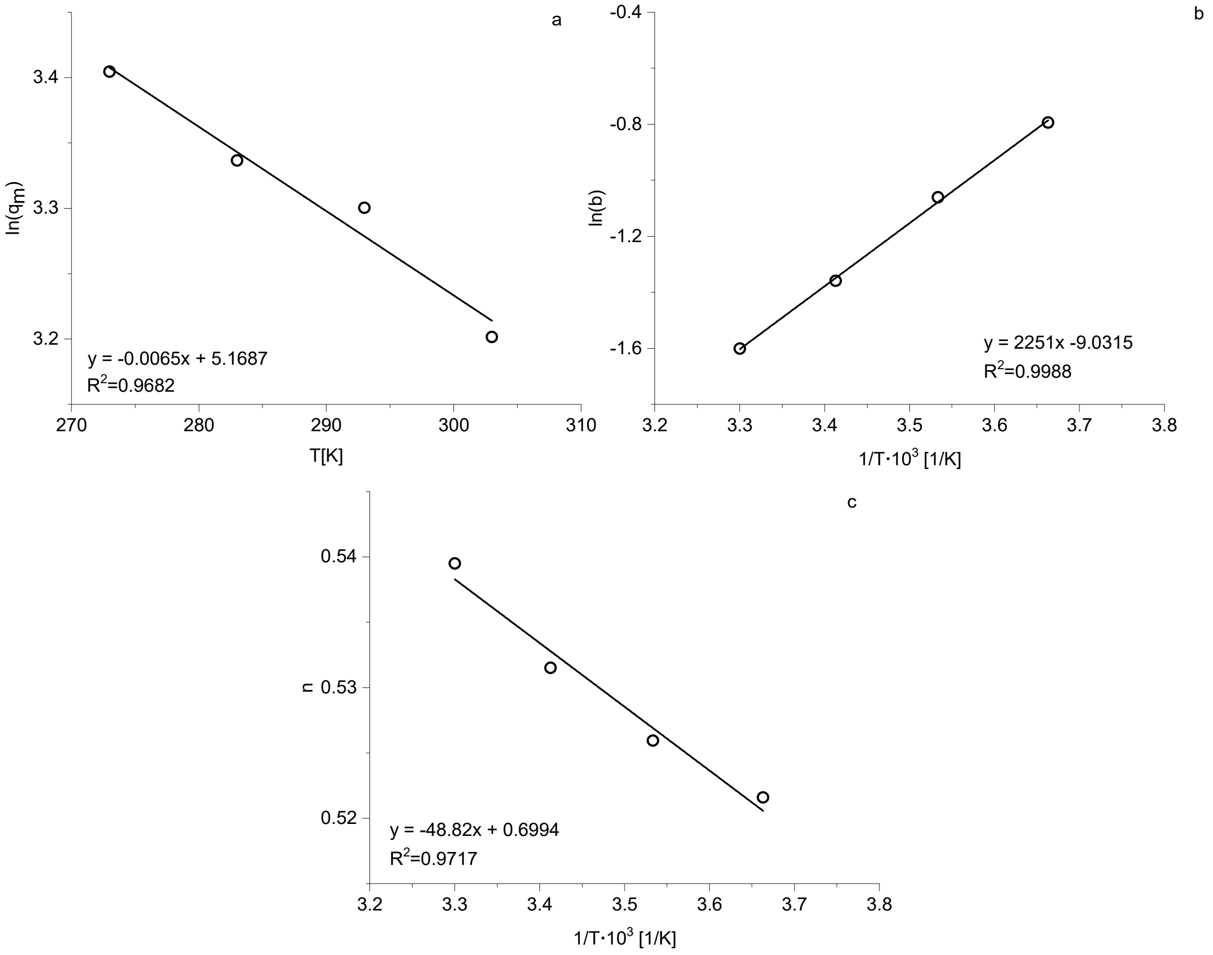

Figure S9. The plots: (a) ln(qm) versus T, (b) ln(b) versus 1/T, and (c) n versus 1/T applied to the calculation of the Toth parameters in equations (1–4) for M-KOH

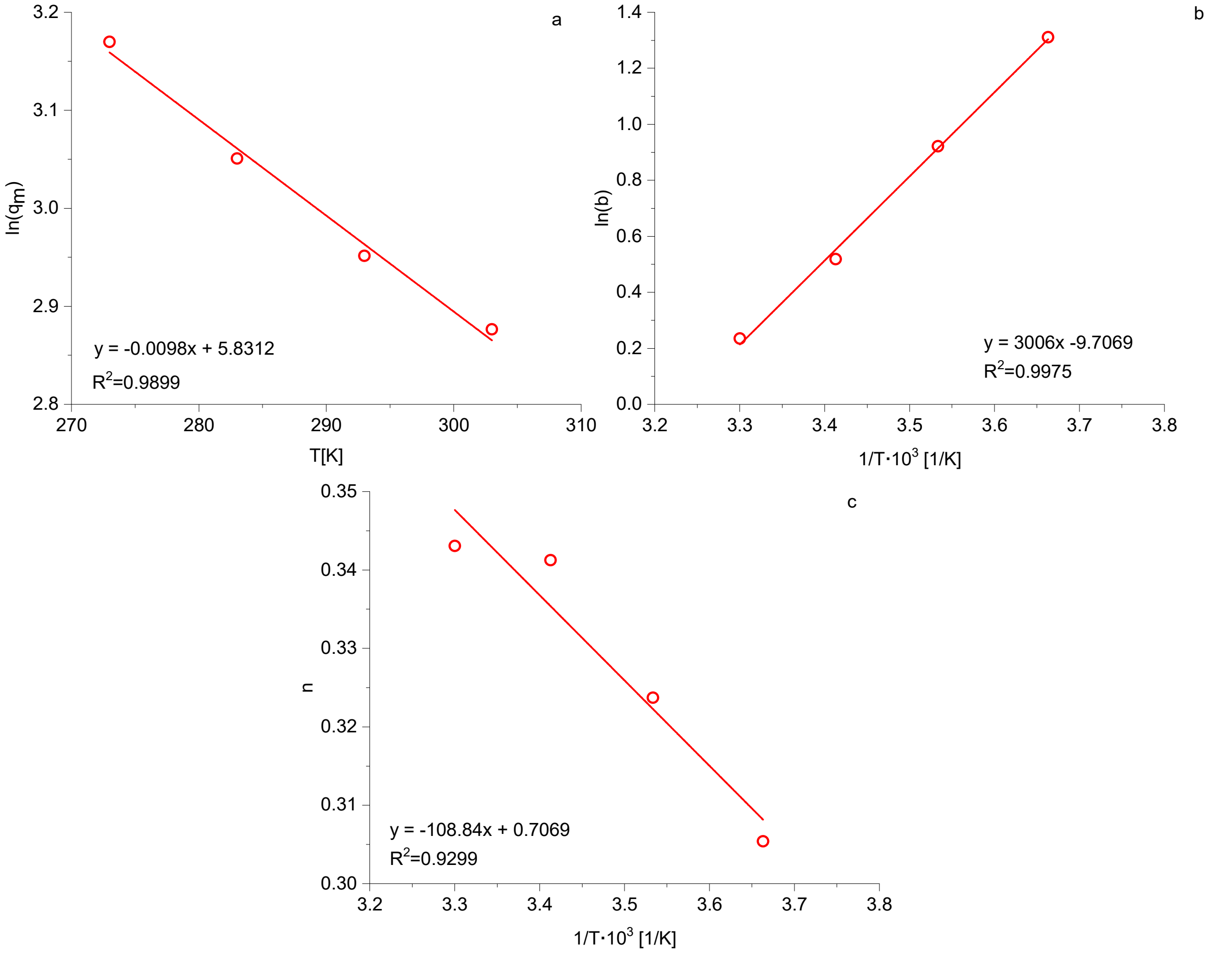

Figure S10. The plots: (a) ln(qm) versus T, (b) ln(b) versus 1/T, and (c) n versus 1/T applied to the calculation of the Toth parameters in equations (1–4) for M-H2SO4

Table S3. The parameters of the Toth temperature depended on equations for M-KOH and M-H2SO4 activated carbons

Parameter	M-KOH	M-H2SO4	Unit
Q	18715	24992	J/mol
b_0	0.228	1.463	bar^{-1}
n_0	0.521	0.308	
α	0.179	0.399	
q_{m0}	30.19	23.55	mmol/g
χ	1.761	2.672	

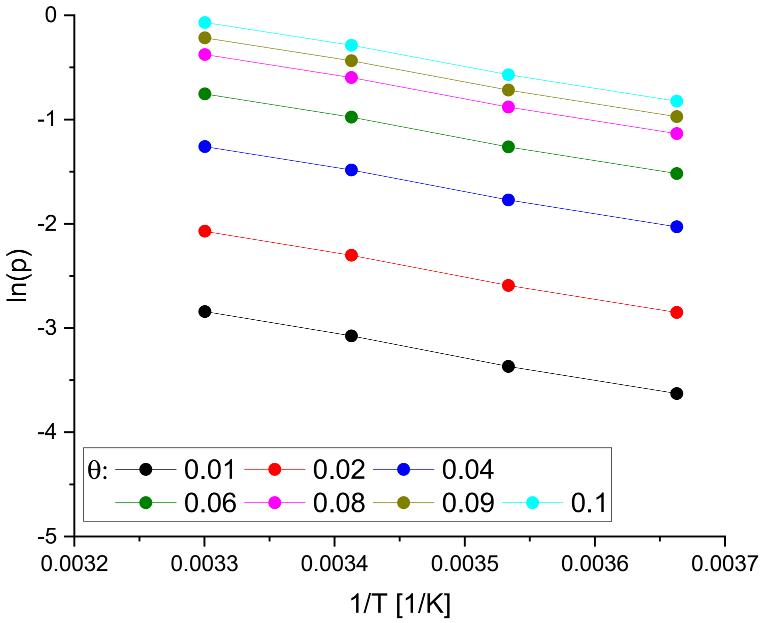

Figure S11. The plot of the function ln(p) vs 1/T for different surface coverage for M-KOH

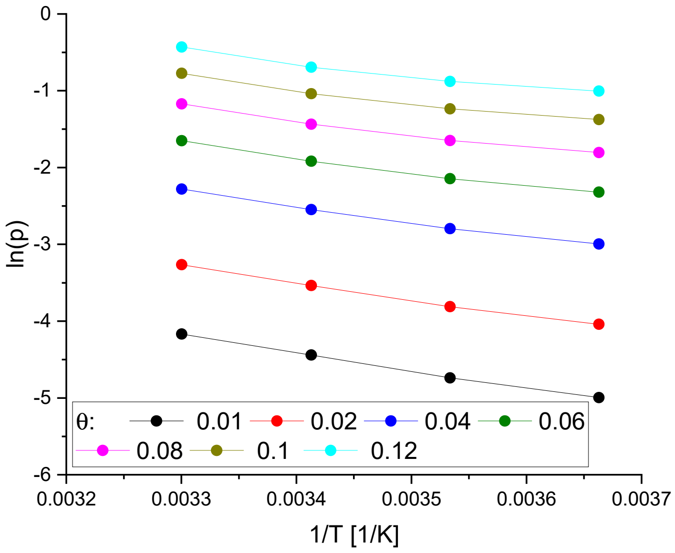

Figure S12. The plot of the function ln(p) vs 1/T different surface coverage for M-H2SO4

The isosteric heat of adsorption:

$$E_{iso} = -R\left(\frac{\partial \ln (p)}{\partial\left(\frac{1}{T}\right)}\right)_{\theta} \qquad \text{(S1)}$$

Linear form of the equation (S1)

$$\ln(p)_{\theta} = -\frac{E_{iso}}{R}\frac{1}{T} + C \qquad \text{(S2)}$$
